# Clinical Characteristics and Outcomes of Severe Pneumonia in Children Under 5 Years Old With and Without Adenovirus Infection in Guangzhou

**DOI:** 10.3389/fped.2021.599500

**Published:** 2021-11-15

**Authors:** Lingling Zheng, Weiyao Liao, Feng Liang, Kuanrong Li, Ling Li, Huiying Liang

**Affiliations:** ^1^Clinical Data Center, The Guangzhou Women and Children's Medical Center, Guangzhou Medical University, Guangzhou, China; ^2^Guangdong Provincial People's Hospital, Guangdong Academy of Medical Sciences, Guangzhou, China; ^3^Department of Nutrition, School of Public Health, Sun Yat-sen University, Guangzhou, China; ^4^The Guangdong Provincial Children's Medical Research Center, Guangzhou, China

**Keywords:** pediatric lung disease, adenovirus, pneumonia, respiratory infection, clinical epidemiology

## Abstract

**Objectives:** To identify the differences of clinical characteristics and outcomes of severe pneumonia in children under 5 years old with and without adenovirus infection.

**Methods:** A retrospective cohort study was conducted in three pediatric hospitals in Guangzhou, China. In total, 1,595 children under the age of 5 with WHO-defined severe pneumonia had adenovirus testing performed between January 1, 2009 and December 31, 2019. Demographics, complications, the first routine laboratory findings, therapeutic records, and clinical outcome were collected from electronic medical records. We compared characteristics of children with and without adenovirus infection.

**Results:** Adenovirus was detected in 75 (4.7%) out of 1,595 children with severe pneumonia. Cases with adenovirus infection were more likely to be boys (74.7 vs. 63.0%), older than 1 year old (78.7 vs. 25.1%), but less likely to have mixed virus infections (25.3 vs. 92.9%) and combined with cardiovascular disease (12.0 vs. 39.7%), and had more abnormal laboratory results than cases without adenovirus infection. Antiviral therapy (4.9%) was rarely used in children with severe pneumonia, but antibiotic therapy (65.3%) was commonly used, especially in cases with adenovirus infection (91.9%). Children infected with adenovirus (9.3 vs. 2.5%) were also hospitalized longer and had a higher mortality within 30 days of hospitalization.

**Conclusions:** Children with severe pneumonia under 5 years old with adenovirus infection had more abnormal laboratory findings and more severe clinical outcomes than cases without adenovirus infection. More attention should be focused on the harm caused by adenovirus infection.

## Introduction

Pneumonia is a leading cause of hospitalization and death among children under 5 years old worldwide, accounting for 15% of all childhood deaths (1). Adenovirus is commonly detected in pediatric severe pneumonia cases, and severe pneumonia can cause chronic complications. Adenoviruses have a worldwide distribution, and should not be ignored as they can cause severe and fatal pneumonia (2). In 2014, the USA reported an increasing number of adenovirus detections from hospitalized patients with severe respiratory infections (3). Studies have identified adenoviruses as the main pathogens associated with severe childhood pneumonia (4–6). And severe pneumonia with adenovirus infection is getting more and more attention around the world (7, 8).

Many previous studies focus on the serotypes of adenovirus and their clinical manifestations because some serotypes are associated with specific clinical manifestations (3, 9–11). However, due to the high cost and lagging test results, clinicians can only detect the type of pathogen by PCR, and it is not possible for them to further detect the serotypes of particular pathogens, except for researchers (12). Adenovirus, as one of the most common viruses isolated from young children with febrile respiratory illness, has no identifiable clinical manifestations, which also leads to challenges for clinicians. In addition, data regarding epidemiology, clinical features, laboratory findings, and outcomes of severe pneumonia among adenovirus infections remain limited.

In this retrospective cohort study, we compared the differences of clinical characteristics and outcomes of severe pneumonia in children under 5 years old with and without adenovirus infection to confirm whether the prognosis of severe pneumonia with adenovirus infection was worse.

## Materials and Methods

A retrospective cohort study was undertaken by using 10 years of electronic health records data from between January 1, 2009 and December 31, 2019 from three pediatric hospitals in Guangzhou, Guangdong Province, China. All pediatric patients were included depending on if they met the inclusion criteria as follows: <5 years old; clinical symptoms suggestive of pneumonia, acquired outside of the hospital or <48 h after hospital admission; met severe pneumonia criteria defined by WHO, with at least one of the following danger signs: persistent vomiting, convulsions, lethargy, no oral intake, stridor, or *severe* malnutrition; took the test for adenovirus infection in the early stage of admission; and were discharged from hospital or died at hospital. And the exclusion criteria were as follows: cases with substantial missing data; and cases without the result of an adenovirus test. The diagnosis of pneumonia in children is based on a physician's diagnosis of bronchitis, bronchiolitis, pneumonia, or any combination of the three, based on X-ray results (13). A total of 1,595 severe pneumonia patients with adenovirus viral detection were included in this analysis.

We collected demographics, complications, the first routine laboratory findings, therapeutic records, and clinical outcome from electronic medical records. Laboratory examinations included complete blood counts, viral testing, and biochemistry to monitor liver, myocardial, and renal functions. Clinical outcomes were 30-day mortality in hospital and hospital length of stay (LOS) in days. All children admitted to the hospital had their respiratory tract samples tested by an RT-PCR assay or rapid antigen test for common pneumonia pathogens like syncytial virus, adenovirus, influenza virus A and B, parainfluenza, bocavirus, people partial pulmonary virus, rhinovirus, and mycoplasma on the same day or the next day. Samples included sputum, throat swabs, or bronchoalveolar lavages (only 10 samples were collected from bronchoalveolar lavages), which were obtained during routine clinical practice. Blood for cultures was obtained from 739 patients with a temperature of ≥38.5°C within 48 h of admission.

Cases with adenovirus infection were considered if one of the following criteria was met: (1) detection of adenovirus in sputum, throat swabs, or bronchoalveolar lavages by RT-PCR; (2) positive antigen in the adenovirus antigen test. Other cases were considered as cases without adenovirus infection. Co-infection was defined as detection of more than one pathogen including viral, bacterial, or atypical pathogens.

This study was approved by the Ethics Committee of Guangzhou Women and Children's Medical Center, which confirmed the need for signed informed consent from the participants. The patients and their next of kin were informed of their inclusion into the database and could decline participation.

### Statistical Analysis

All patients were divided into the following two groups: the severe pneumonia with adenovirus infection group and severe pneumonia without adenovirus infection group. We compared characteristics of children with and without adenovirus infection, including age, gender, presence and types of complication, hospitalization time, laboratory findings, treatments, and outcomes of severe pneumonia. Categorical variables were summarized by frequencies and percentages. Continuous variables were expressed as median [IQR] or means ± SD whichever was appropriate. We used the χ2 test or Wilcoxon rank-sum tests for bivariate comparisons. All tests were two-sided and *p* < 0.05 were considered statistically significant. Associations between ADV infection and main clinical outcome were assessed by Cox proportional hazards regression for time to death in hospital within 30 days. And statistical analyses were performed with the R software (Version 2.8.1).

## Results

### Clinical Characteristics

**Figure 1:**
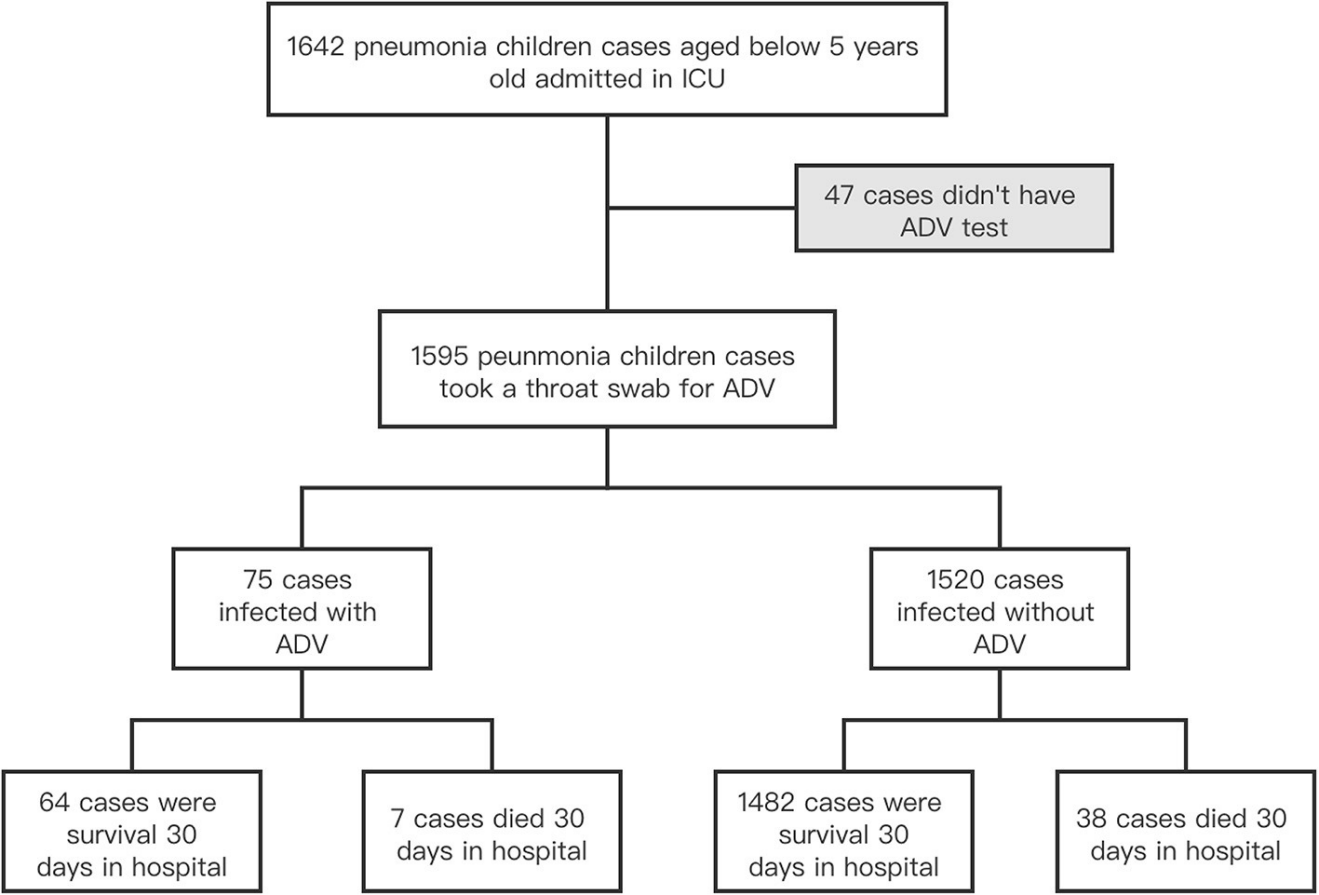
The study flowchart. ADV, adenovirus.

Of the 1,595 severe pneumonia children enrolled in this study, 75 (4.7%) had an adenovirus infection. The fatality rate of cases with adenovirus infection (9.3%) was higher than cases without adenovirus infection (2.5%) (Table 1, Figure 1). More than two-thirds of the severe pneumonia pediatric cases were <1 year old, but most cases with adenovirus infection were older than 1 year old. Severe pneumonia children with adenovirus infection were more likely to be boys, older than 1 year old, and infected with a combination of other microorganisms, but less likely to have mixed virus infection and co-occurring cardiovascular disease than cases without adenovirus infection (Table 1).

**Table 1:** Characteristics of 1,595 children with severe pneumonia with and without ADV infection.

**Characteristics**	**Total, *n* = 1,595**	**With ADV infection, *n* = 75**	**Without ADV infection, *n* = 1,520**	***P***
Gender, *n* (%)				0.041
Male	1,014 (63.6)	56 (74.7)	958 (63.0)	
Female	581 (36.4)	19 (25.3)	562 (37.0)	
Age at diagnosis (month), median [IQR]	1 (1, 3)	10 (5, 16)	1 (1, 3)	<0.001
Age Category, *n* (%)				<0.001
<1 year	1154 (72.4)	16 (21.3)	1138 (74.9)	
1–4 years	441 (27.6)	59 (78.7)	382 (25.1)	
Co-infection	467 (29.3)	19 (25.3)	448 (29.5)	<0.001
No pathogen infection	1,072 (67.2)	-	1072 (70.5)	
Viral infection				<0.001
Single infection	88 (16.8)	56 (74.7)	32 (7.1)	
Mixed infection	435 (83.2)	19 (25.3)	416 (92.9)	
Comorbidities				
Cardiovascular disease	612 (38.4)	9 (12.0)	603 (39.7)	<0.001
Gastrointestinal disease	268 (16.8)	12 (16.0)	256 (16.8)	0.85
Hepatic and bile disease	127 (8.0)	6 (8.0)	121 (8.0)	0.99
Renal damage	32 (2.0)	1 (1.3)	31 (2.0)	0.67
Cerebral damage	271 (17.0)	9 (12.0)	262 (17.2)	0.23
Immunodeficiency	11 (0.7)	0 (0.0)	11 (0.7)	0.46
Malnutrition	100 (6.3)	4 (5.3)	96 (6.3)	0.73
G6PD deficiency	52 (3.3)	2 (2.7)	50 (3.3)	0.77
Blood disease	58 (3.6)	5 (6.7)	53 (3.5)	0.15

### Laboratory Tests

In general, cases with adenovirus infection had more abnormal laboratory results than cases without adenovirus infection. Blood routine testing and blood biochemistry showed that all children with severe pneumonia had a higher lymphocyte count and hemoglobin, and lower albumin, but these three items in cases with adenovirus infection were lower than in cases without adenovirus infection. Besides, cases with adenovirus infection had lower lymphocyte levels (5.49 ± 0.92) and higher neutrophil levels (3.75[2.22, 6.47]) than cases without adenovirus infection (lymphocytes, 7.51 ± 0.6; neutrophils 4.01[2.60, 6.56]) (Table 2). In the coagulation function test, all cases had longer APTT and higher D-dimer which indicated that cases with adenovirus infection had worse coagulation function than cases without adenovirus infection (Table 2). In other laboratory findings, children with severe pneumonia had higher LDH, while cases with adenovirus infection (746[464, 1,378] U/L) were higher than cases without adenovirus infection (326[278, 407] U/L). In addition, cases with adenovirus infection had abnormal glucose (6.25[5.2, 7.5] mmol/L) and CRP (13.45[3.9, 34.8] mg/L) levels (Table 2).

**Table 2:** Laboratory findings of 1,595 children with severe pneumonia with and without ADV infection.

**Characteristics**	**Reference**	**With ADV infection**	**Without ADV infection**	***P***
**Blood routine**				
Leucocyte count, ×109/L	5.0–12.0	8.52 ± 5.56	11.95 ± 7.82	<0.001
Lymphocyte count, ×109/L	1.55–4.80	5.4 ± 9.92 ↑	7.45 ± 10.64 ↑	0.026
Neutrophil count, ×109/L	2.0–7.2	3.75 [2.22, 6.47]	4.01 [2.60, 6.56]	<0.001
Lymphocytes, %	40–60	32 [23, 46] ↓	46 [32, 56]	<0.001
Neutrophils, %	31–40	60[44,71] ↑	40[29,55]	<0.001
Platelets, %	0.1–0.5	0.27 [0.18, 0.42]	0.40 [0.31, 0.49]	<0.001
Hemoglobin, g/L	135–195	328.14 ± 32.31 ↑	335.26 ±1 5.02 ↑	<0.001
**Blood biochemistry**				
Albumin, g/L	40–55	33.38 ± 6.38 ↓	37.26 ± 4.34 ↓	<0.001
**Coagulation function**				
APTT, s	11–15	43.63 ± 15.89 ↑	43.54 ± 7.31 ↑	<0.001
PT, s	11-15	13.68 ± 2.46	13.97 ± 2.68	0.58
D-dimer, mg/L	<0.5	2.64 [1.38, 4.65] ↑	1.07 [0.54, 2.07] ↑	<0.001
**Other Laboratory findings**				
ALT, U/L	7–40	22.0 [15.0,32.5]	20.0 [13.0, 30.3]	<0.001
AST, U/L	5–60	55.0 [15.0,32.5]	35.0 [28.0, 49.0]	<0.001
Total bilirubin, μmol/L	2–17	3.8 [2.2,5.9]	24.85 [7.40, 72.0] ↑	>0.99
LDH, U/L	159–322	746 [464,1378] ↑	326 [278, 407] ↑	<0.001
BUN, mmol/L	2.1–7.1	3.91 ± 4.21	3.09 ± 2.69	0.0017
Scr, μmol/L	18–97	22.88 ± 8.9	25.4 ± 30.37	0.70
CK, U/L	45–390	114 [74, 226]	113 [76, 195]	<0.001
Glucose, mmol/L	4.1–5.9	6.25 [5.2, 7.5] ↑	5.5 [4.8, 6.5]	<0.001
CRP, mg/L	≤ 8.2	13.45 [3.9, 34.8] ↑	2.4 [0.6, 10.6]	<0.001

*Data in the table are median [IQR], mean ± SD. P values are derived from χ^2^ test*.

*↑: the mean of the results was over the reference of normal value*.

*↓: the mean of the results was under the reference of normal value*.

*APTT, activated partial thromboplastin time; PT, prothrombin time; ALT, alanine aminotransferase; AST, aspartate aminotransferase; BUN, blood urea nitrogen; Scr, serum creatinine; CK, creatine kinase; LDH, lactate dehydrogenase; CRP, C-reactive protein*.

### Therapeutic and Clinical Outcomes

A total of 1586 severe pneumonia pediatric cases who described their treatment processes, and 9 cases who did not (0.56%) were randomly distributed in cases with and without adenovirus infection. Antiviral therapy (like ribavirin or oseltamivir) was rarely used in children with severe pneumonia, but antibiotic therapy (like azithromycin or cefoperazone sodium) was commonly used, especially cases with adenovirus infection (91.9%). Compared with cases without adenovirus infection, more cases with adenovirus infection were treated with corticosteroids and immunoglobulin, and oxygen therapy was used in 82.4% of cases with adenovirus infection but only 51.5% of cases without ADV infection. Besides, more cases with adenovirus infection were treated with invasive mechanical ventilation.

**Figure 2:**
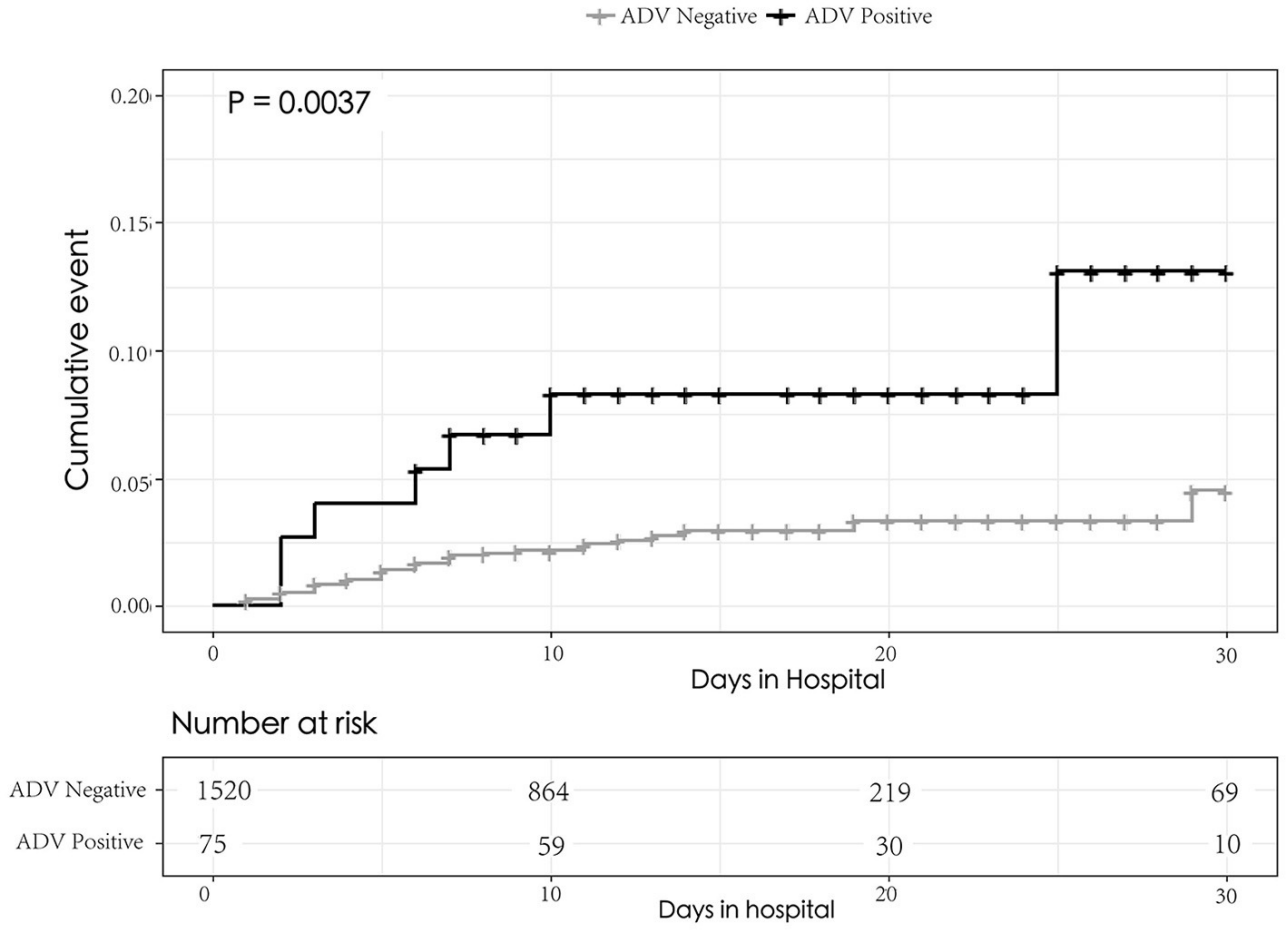
Kaplan–Meier graphs of the probability of death for 30 days in hospital between the sever pneumonia children with and without ADV infection. The curves were compared using the log rank test.

During hospitalization, 64.0% of cases with adenovirus infection suffered respiratory failure, which was higher in cases without adenovirus infection (24.7%). And cases with adenovirus infection also had a longer duration at hospital and a higher in-hospital 30-day mortality rate (9.3%) than cases without adenovirus infection (2.5%). A Cox model result showed that children with severe pneumonia with adenovirus infection had higher risk for in-hospital 30-day mortality (hazard ratio: 3.1; CI: 1.4–7.1) (Table 3, Figure 2).

**Table 3:** Therapeutic and clinical outcome of 1,586 children with severe pneumonia with and without ADV infection.

**Characteristics**	**ALL, *n* = 1,586**	**With ADV infection, *n* = 74**	**Without ADV infection, *n* = 1,512**	***P***
**Therapeutic features**				
Antiviral therapy	78 (4.9)	6 (8.1)	72 (4.8)	0.17
Antibiotic therapy	1036 (65.3)	68 (91.9)	968 (64.0)	<0.001
Use of corticosteroid	672 (42.4)	66 (89.2)	606 (40.1)	<0.001
Use of adrenaline	643 (40.5)	66 (89.2)	577 (38.2)	<0.001
Use of other corticosteroid	279 (17.6)	36 (48.6)	243 (16.1)	<0.001
Use of immunoglobulin	319 (20.1)	47 (63.5)	272 (18.0)	<0.001
Oxygen therapy	840 (53.0)	61 (82.4)	779 (51.5)	<0.001
Invasive mechanical ventilation	405 (25.5)	44 (59.5)	361 (23.9)	<0.001
**Clinical outcome**				
Respiratory failure	424 (26.6)	48 (64.0)	376 (24.7)	<0.001
Duration at hospital (day), median [IQR]	11 (8, 16)	17 (10, 24)	10 (8, 15)	<0.001
In-hospital 30-day mortality	45 (2.8)	7 (9.3)	38 (2.5)	<0.001

*Data in the table are median (IQR), mean (SD), n (%), or n/N (%), where N is the total number of patients with relevant data. P values are derived from χ^2^ test or Fisher's exact test*.

*RR, relative risk; NA, not available; NE, not estimable*.

## Discussion

Children with severe pneumonia with adenovirus infection had more abnormal laboratory findings and more severe clinical outcomes than cases without adenovirus infection, including suffering more respiratory failure, longer LOS, and higher mortality.

In our study, the median age of children with severe pneumonia was 10 months, and children were older than those without adenovirus infection. The study in Singapore showed most pediatric patients infected with adenovirus were below 2 years old, and another study showed that the median age of children with adenovirus pneumonia in Malaysia was 1.08 years, which was similar to ours (14, 15). It is supposed that newborns who have immunity from their mothers can fight off adenovirus infection, but as times goes on, the immune defense from mothers fades, and as their own immune systems are not yet fully mature, children around the age of one are vulnerable to adenovirus infection. It has been confirmed that adenovirus mainly infects immunocompromised people, so children aged around 1 year may be at higher risk of adenovirus infection. However, fatal cases have also been reported among newborns, therefore, more evidence is needed to support this hypothesis (16, 17).

A total of 25.3% of children with severe pneumonia with adenovirus infection were co-infected by other microorganisms, like RSV, influenza virus, or parainfluenza viruses. A study in Turkey also showed that 12.1% of children with respiratory infection were found to have at least two virus infections, and the most common viral agent was HRV followed by adenovirus (18). Only 2.1% of cases without adenovirus infection were found to have more than one virus. According to this finding, we supposed that children infected with adenovirus have a weaker immune system than children infected without adenovirus.

More children infected without adenovirus had cardiovascular disease (39.7%) compared with adenovirus-infected children (12.0%), while there was no statistical significance for other diseases. However, a Taiwanese study suggested that prematurity and congenital heart diseases do not show statistical significance for adenovirus pneumonia, but they are associated with disease severity (19). Underlying neurological disease and respiratory disease were more prevalent in severe adenovirus infection and pneumonia. Tsou showed that patients with underlying conditions, especially neurologic diseases, were more likely to suffer from adenovirus infections (20). One study about risk factors associated with mortality of children with pneumonia reported that malnutrition was the most common factor related with fatality (21). The study of Zampoli et al. in South Africa reported that 34.0% of children with adenovirus-associated pneumonia were malnourished (22). But in our study, children with adenovirus infection had a lower risk of being co-infected with other diseases than cases without adenovirus infection.

Children with severe pneumonia with adenovirus infection had more abnormal laboratory results than cases without adenovirus infection. Higher levels of LDH indicated more severe injury and reflected the possibility of hepatitis (23). Severe pneumonia cases had a high serum level of LDH, which was consisted with the Erez study, while the LDH level of cases with adenovirus infection was twice as high as cases without adenovirus infection. Lai et al. had similar results in both the serum and pleural fluid levels in severe adenovirus respiratory infection (24, 25). Wu et al. suggested that a high serum level of LDH and a low lymphocyte count could be used as predictors for the severity of adenovirus respiratory infection in children (26). On the contrary, both severe pneumonia cases with and without adenovirus infection in our results showed a high lymphocyte count, but only cases with adenovirus infection had a low percentage of lymphocytes. It means that lymphocytes might not be an appropriate predictor for severity of adenovirus infection in children.

In addition, children with severe pneumonia had a low level of albumin and a long coagulation time. Especially, children with pneumonia with ADV infection had a lower level of albumin (33.386 ± 0.38 g/L) and worse coagulation function (APTT, 43.631 ± 5.89). The adenovirus might attack the hematopoietic system or immune system which influences the level of albumin and coagulation function. The study of Miao et al. showed that children with severe adenovirus pneumonia with low serum albumin may have poor prognosis (27). Cases with adenovirus infection had higher levels of CRP, which was consistent with Chen's study, revealing that elevated CRP levels were common in adenovirus infection, even without superimposed bacterial infection (28). Specially, cases with adenovirus infection showed a high level of serum glucose. We supposed that the adenovirus might damage children's Langerhans β cells, but more evidence is needed to support this hypothesis.

Only a few children with severe pneumonia received antiviral therapy, but the majority of them received the antibiotic therapy, especially the cases with adenovirus infection. The benefits of treatment with antiviral therapy for severe adenovirus pneumonia are still not confirmed (29). Due to the limitation of test methods, detecting all concomitant bacterial infections is difficult (14), and only the impact of bacterial co-infection on disease severity and mortality has been reported in patients with viral infection (30, 31). So, most clinicians use antibiotic drugs based on clinical experience. In some randomized controlled trials and observational studies, rapid recognition of viruses was not associated with reducing antibiotic use (32, 33). Additionally, we found more cases with adenovirus infection were treated by corticosteroids like adrenaline as the first aid medicine, oxygen therapy, and invasive mechanical ventilation, which revealed that children with adenovirus infection might have more serious disease. It is worth noting that a randomized clinical trial found that among patients with severe pneumonia the acute use of corticosteroids could reduce treatment failure compared with placebo. Therefore, they suggested the use of corticosteroids as adjunctive treatment for patients with severe pneumonia (34).

A total of 64.0% of children with severe pneumonia with adenovirus infection suffered respiratory failure on admission, which was more than double the figure of cases without adenovirus infection. They also had a longer duration in hospital. In our result, the 30-day mortality in hospital among cases with adenovirus infection was 9.3%, consistent with Wu's study, higher than 2.5% of cases without adenovirus infection (28). But it is lower than another study in 415 hospitalized children under 6 years of age with ALRI caused by adenovirus in Argentina from 1988 to 2005 who had a 15% mortality (9). The high risk in mortality with adenovirus infection suggested that adenovirus surveillance programs should be in place to monitor peaks in infection rates (8).

Like most retrospective epidemiological studies, data were often incomplete and analyses may be biased. We were unable to detect a common virus to compare adenovirus infection with another viral infection. Therefore, the findings in our study should be interpreted with caution.

## Conclusion

Children with severe pneumonia with adenovirus infection had more abnormal laboratory findings and more severe clinical outcomes than cases without adenovirus infection. More attention needs to be focused on the harm caused by adenovirus infection.

## Data Availability Statement

The raw data supporting the conclusions of this article will be made available by the authors, without undue reservation.

## Ethics Statement

The studies involving human participants were reviewed and approved by the Ethics Committee of Guangzhou Women and Children's Medical Center. Written informed consent to participate in this study was provided by the participants' legal guardian/next of kin. Written informed consent was obtained from the individual(s), and minor(s)' legal guardian/next of kin, for the publication of any potentially identifiable images or data included in this article.

## Author Contributions

All authors listed have made a substantial, direct and intellectual contribution to the work, and approved it for publication.

## Funding

This work was supported from the National Natural Science Foundation of China (Grant no. 81401755).

## Conflict of Interest

The authors declare that the research was conducted in the absence of any commercial or financial relationships that could be construed as a potential conflict of interest.

## Publisher's Note

All claims expressed in this article are solely those of the authors and do not necessarily represent those of their affiliated organizations, or those of the publisher, the editors and the reviewers. Any product that may be evaluated in this article, or claim that may be made by its manufacturer, is not guaranteed or endorsed by the publisher.
